# Hematological consequences of malaria infection in mice previously treated for visceral leishmaniasis

**DOI:** 10.12688/wellcomeopenres.16629.1

**Published:** 2021-04-16

**Authors:** Gulab Fatima Rani, Helen Ashwin, Najmeeyah Brown, Ian S. Hitchcock, Paul M. Kaye

**Affiliations:** 1 Hull York Medical School, University of York, UK, York, N. Yorks, Yo10 5DD, UK; 2 Department of Biology, University of York, UK, York, N. Yorks, Yo10 5DD, UK

**Keywords:** visceral leishmaniasis, malaria, mouse models, hematology, chemotherapy, coinfection

## Abstract

**Background**: Polyparasitism is commonplace in countries where endemicity for multiple parasites exists, and studies in animal models of coinfection have made significant inroads into understanding the impact of often competing demands on the immune system. However, few studies have addressed how previous exposure to and treatment for one infection impacts a subsequent heterologous infection.

**Methods**: We used a C57BL/6 mouse model of drug-treated *Leishmania donovani* infection followed by experimental *Plasmodium chabaudi* AS malaria, focusing on hematological dysfunction as a common attribute of both infections. We measured parasite burden, blood parameters associated with anemia and thrombocytopenia, and serum thrombopoietin. In addition, we quantified macrophage iNOS expression through immunohistological analysis of the liver and spleen.

**Results**: We found that the thrombocytopenia and anemia that accompanies primary *L. donovani* infection was rapidly reversed following single dose AmBisome® treatment, along with multiple other markers associated with immune activation (including restoration of tissue microarchitecture and reduced macrophage iNOS expression). Compared to naive mice, mice cured of previous VL showed comparable albeit delayed clinical responses (including peak parasitemia and anemia) to *P. chabaudi* AS infection. Thrombocytopenia was also evident in these sequentially infected mice, consistent with a decrease in circulating levels of thrombopoietin. Architectural changes to the spleen were also comparable in sequentially infected mice compared to those with malaria alone.

**Conclusions**: Our data suggest that in this sequential infection model, previously-treated VL has limited impact on the subsequent development of malaria, but this issue deserves further attention in models of more severe disease or through longitudinal population studies in humans.

## Introduction

In countries where multiple parasites are endemic, polyparasitism – the presence of multiple species of parasites in the same individual – is commonplace. Polyparasitism involving various bacterial, viral and parasitic diseases, including malaria, schistosomiasis and other helminthoses, tuberculosis, and HIV is well documented and can lead to synergistic or antagonistic clinical and immunological effects ^1– 4^. In experimental models of leishmaniasis, a number of previous studies have also addressed the issue of coinfection. For example, in mice coinfected with *Leishmania donovani* and *Schistosoma mansoni,* granulomatous inflammation in the liver progressed normally, although *L. donovani*-induced granulomas found nested within the granulomas induced by *S. mansoni* eggs failed to develop normally ^5^. Experimental visceral leishmaniasis (VL) was also shown to confer enhanced protection against *Streptococcus pneumoniae* sepsis ^6^. Similarly, in experimental models of malaria as a concurrent or superimposed infection, aggravating or protective responses were noticed ^7– 10^, suggestive of the role of immune dysregulation.

In areas where human VL and malaria are endemic, the prevalence of coinfection can be high. For example, one cross-sectional retrospective study in Sudan indicated that up to 31% of hospitalized VL cases had concomitant malaria ^11^. Similarly, cross-sectional studies in Ethiopia provided a prevalence estimate for coinfection of 2–4% ^12, 13^. In contrast to the wealth of data on coinfection, the question of whether previous history of VL impacts on subsequent episodes of malaria has not been documented. Similarly, risk factors for VL recurrence have been identified including persistent splenomegaly, poor hematological response and VL/HIV coinfection ^14– 17^ but the incidence of post-treatment (post-Rx) secondary infections with malaria has not been reported.

A number of recent studies suggest that primary systemic infections may have significant long-term impacts on immunological and hematological health. For example, experimental VL has been shown to impact hematopoiesis by driving premature hematopoietic stem cell exhaustion and emergency hematopoiesis may impact myeloid cell function ^18– 21^. Similarly, dysfunctional medullary erythropoiesis leading to anemia has also been reported in experimental VL ^22^. Immunosuppression due to dysregulated immune responses, lack of early diagnosis and unavailability of effective treatment could make these patients more at risk of developing concomitant or sequential infections.

Here, we describe a study aimed at addressing the question of whether mice infected with *L. donovani* and then treated with an effective therapeutic regimen (AmBisome *®*) have altered clinical and parasitological outcomes to a subsequent *Plasmodium chabaudi* (AS) malaria infection. We focus on two aspects of immune dysfunction associated with both primary VL and malaria, namely changes in tissue architecture and hematological profile.

## Methods

### Ethical approval

Ethical approval for the study was obtained from the Animal Welfare and Ethical Review Board of the Department of Biology, University of York, York. All procedures were performed under the authority of a UK Home Office Project License (P49487014). All efforts were made to ameliorate harm by adherence to defined clinical endpoints (including physical and clinical condition) and daily monitoring for any unexpected behaviour. No unexpected adverse events were recorded during this study.

### Sample

C57BL/6 mice bred and maintained at the Biological Services Facility (BSF), University of York were originally sourced from Envigo (Huntingdon, UK). Genetic profiling of mice from the colony using microsatellite markers was conducted at Surrey Diagnostics Ltd (Cranleigh, UK), confirming identity to C57BL/6J at 27 microsatellite markers. Two mice (of four tested) had an additional allele at marker 138 (192bp) one mouse had an additional allele at marker 134 (112bp) and two mice had an additional allele at marker 144 (195bp). Mice were kept in individual ventilated cages at 20–21°C and 56% humidity under specific pathogen-free (SPF) conditions (FELASA 67M and 51M) and provided with food and water *ad libitum* and with cage enrichment. Mice included in experiments were six-eight week old females, of excellent health status and that had not been subject to any genetic manipulation or previous regulated procedures. There were no formal exclusion criteria. To estimate sample size, we used data from Hewitson *et al*. ^23^ for hemoglobin (Hb) concentration and malaria parasitemia ^24^. A sample size of 5 mice per group provides >80% power to detect a 20-25% change when comparing *Leishmania*-infected / drug treated mice with uninfected mice or comparing malaria single-infected mice with sequentially infected mice.

**Figure 1.**
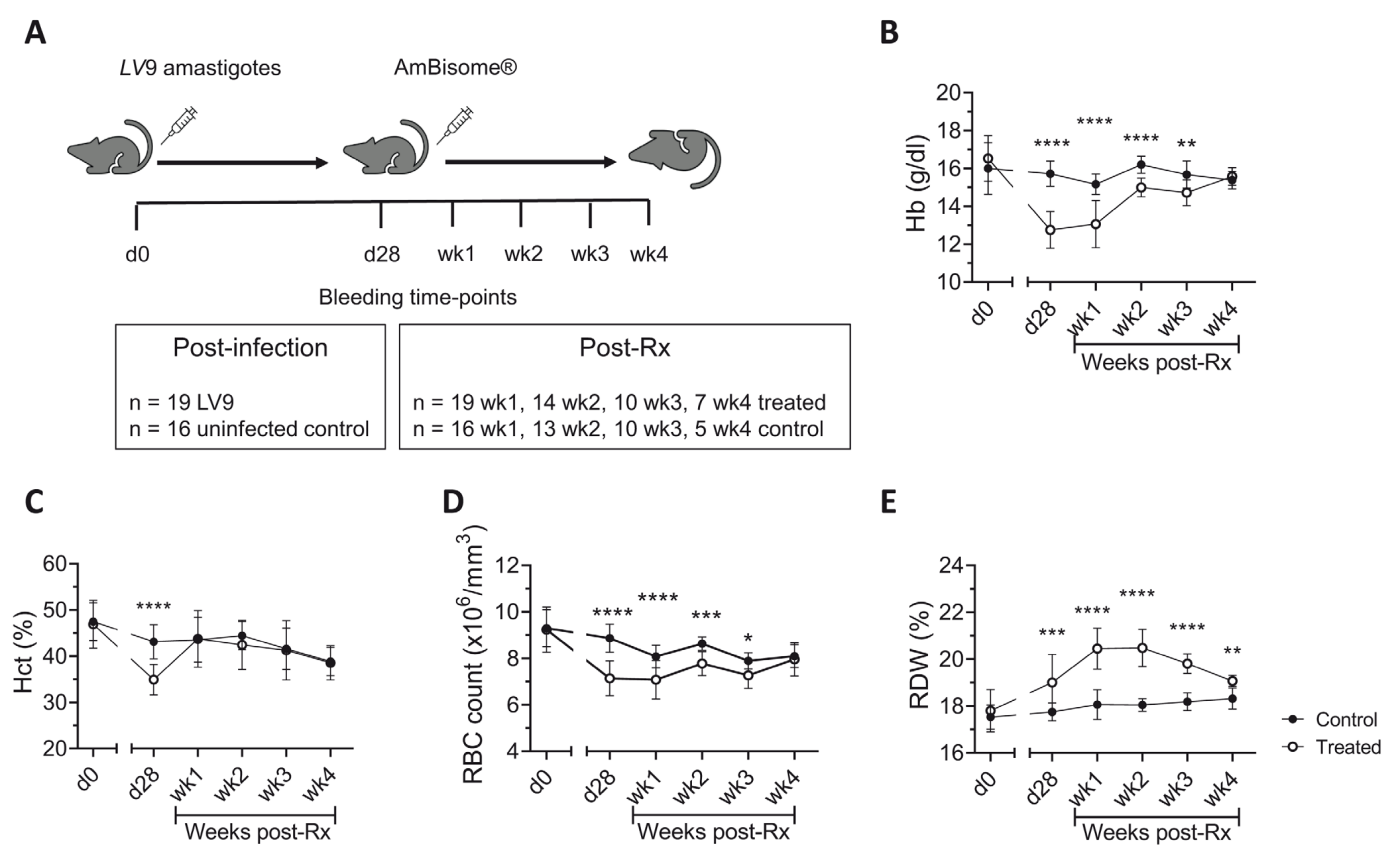
Recovery of red blood cell parameters in drug-treated *L. donovani* infected mice. **A**) C57BL/6 mice were infected with 3 × 10 ^7^ LV9 amastigotes intravenously (i.v) followed by treatment with a single intravenous (i.v) dose (8mg/kg) of AmBisome® at d28 p.i. All mice were bled via lateral tail vein for blood count analysis at the times indicated. **B**– **E**) Hemoglobin (Hb; **B**), Hematocrit (Hct; **C**), RBC count ( **D**) and red cell distribution width (RDW; **E**) were determined on uninfected control and AmBisome® treated mice at d0, d28 p.i and weekly post-Rx for four weeks. Data are representative of a single experimental with n=16 control mice and n=19 LV9 infected/drug-treated mice at d0, d28 and week1, n=13 control and n=14 treated mice at week2, n=10 control and n=10 treated mice at week3, n=5 control and n=7 treated mice at week 4. Data analysed using unpaired t test comparing mean ± SD of uninfected control vs drug-treated mice at each time-point, *, p < 0.05; **, p < 0.01; ***, p < 0.001; ****, p < 0.0001.

***Procedures.*** *For L. donovani* infections, mice were infected once with 3x10 ^7^ amastigotes of an Ethiopian strain of *L. donovani* (LV9) via the intravenous route without anesthesia, and infection allowed to proceed for 28 days prior to any subsequent treatment. As required, *L. donovani*-infected mice were treated with a single dose of AmBisome® (8mg/kg, intravenously, resuspended in sterile 5% dextrose in distilled water; Gilead Sciences International, Ltd, Cambridge, UK) at day 28 post infection (p.i). To assess treatment response in *L. donovani*-infected mice, groups of mice were killed at d28 post infection (p.i.) i.e. prior to AmBisome® treatment and at weeks 1, 2, 3 and 4 post AmBisome® treatment (see Figure 1A for schematic representation).

Malaria infection was established by injection of 1x10 ^5^ infected red blood cells (iRBCs) of the non-fatal *P. chabaudi chabaudi* AS strain (herein referred to as *P. chabaudi*) via the intravenous route without anaesthesia. Mice were kept under a strict 12-hour light-dark cycle to maintain the circadian rhythm of the parasite, critical for the progression of malaria infection. For sequential infection, *L. donovani*-infected mice treated once with AmBisome® as above were rested for four weeks before malaria infection. These mice (designated as VTM mice) were compared to mice with malaria infection only (M mice) and to age-matched uninfected control mice (C mice), *L. donovani*-infected mice that did not receive AmBisome® (VU mice) and AmBisome®-treated *L. donovani*-infected mice not infected with malaria (VT). To assess outcomes in sequential infection, mice were sampled for blood counts at d28 p.i with *L. donovani* and 4 weeks after AmBisome® treatment and for blood counts and parasitemia at days 5, 7, 9, 11, 13, 15 and 18 post *P. chabaudi* infection. Mice in all groups were killed at day 18 p.i. with *P. chabaudi* (see Figure 4A for schematic representation). 

In each experiment, mice were randomly allocated (using Rand function in Microsoft Excel or by drawing lots and ear tagging) to their respective treatment groups. Downstream analysis of blood and tissue was performed blind to treatment group. Randomization and blinding was performed by members of the research team not involved in subsequent analysis. All animals were visually inspected daily for signs of ill health and were within accepted humane endpoints (loss of >20% body weight, piloerection, lethargy). Mice were killed by Home Office approved methods of CO _2_ inhalation or overdose of inhaled anesthetic (Isoflurane) followed by cervical dislocation at d18 after malaria infection.

***Outcome measures***

**Determination of parasite burden**

*Leishmania* parasite burden was determined post-mortem and calculated from counts of Giemsa-stained tissue impression smears and is presented as Leishman-Donovan units (LDUs), where LDU = number of amastigotes/1000 cell nuclei x organ weight (grams). Parasite burdens were determined at the times indicated above. *P. chabaudi* parasitemia was calculated from Giemsa-stained blood smears, using blood sampled at the times given above, where percentage parasitemia = number of iRBCs/total RBCs counted x 100%.

**Blood collection and analysis**

Complete blood count (CBC) analysis was performed using a scil Vet abc Plus+ blood counter (scil animal care company, Dumfries, Scotland, UK) on blood samples collected in EDTA-coated tubes (Microvette CB300 EDTA, Sarstedt, Germany).

The blood parameters analysed includes platelet count, mean platelet volume (MPV), hemoglobin (Hb), Red blood cells (RBC) count, hematocrit (Hct), red blood cell indices (mean cell volume; MCV, mean cell hemoglobin; MCH, mean cell hemoglobin concentration; MCHC, red cell distribution width; RDW), total white blood cell count (WBC) and individual white blood cell counts (neutrophil count, lymphocyte count, eosinophil count, monocyte count).

**Estimation of circulating thrombopoietin (TPO) levels**

Unbound circulating TPO levels were measured in serum samples of experimental and control mice at week 1, 2, 3, 4 post-Rx and d18 post-malaria infection, using a Mouse Thrombopoietin Quantikine ELISA Kit (MTP100; R & D systems, Minneapolis, MN, USA) as per manufacturer’s guidelines. Some samples were omitted from TPO analysis due to assay limitations.

**Tissue histology**

Livers and spleens of infected and control mice were harvested post-mortem at the end of each experiment, as above, and embedded in cryomolds using OCT Tissue-Tek and snap frozen on dry ice. Tissues were kept at -80 ^○^C until needed for further processing. Cryo-embedded tissue sections were cut at a thickness of 8–10μm using CM1900 cryostat (Leica Microsystems, Wetzlar, Germany) and allowed to air dry prior to staining. Tissue sections were fixed with ice-cold acetone for 5 minutes followed by staining with Harris Haematoxylin stain for 10 minutes. Slides were rinsed thoroughly in running water followed by staining with 0.5% Eosin in 95% ethanol. Slides were washed in running tap water and then processed through ethanol solutions for mounting. Staining was carried out at room temperature (RT). Slides were coverslipped with Depex mounting medium (SLS, Nottingham, UK) and left to dry at RT. Images were captured using an AxioScan.Z1 slide scanner (Zeiss, Oberkochen, Germany) at 20x resolution.

**Immunohistochemistry**

Cryo-embedded livers and spleens were sectioned as described above and fixed in ice-cold acetone for 5 minutes after marking the tissue outlines with ImmEDGE™ Hydrophobic Barrier pen (Vector Laboratories Ltd., Peterborough, UK). Slides were washed with wash buffer (0.05% w/v bovine serum albumin; BSA (Sigma-Aldrich, USA) in sterile 1x phosphate-buffered saline (PBS)) followed by blocking with dilution buffer (5% serum in wash buffer) for 30 minutes at RT. Liver sections were incubated with F4/80 AF647 (Host: Rat, Clone: BM8, Dilution: 1:200, Cat no: 123122, BioLegend) for Kupffer cells and/or unconjugated anti-TPO antibody (Host: Rabbit, Clone: EPR14948, Dilution: 1:100, Cat no: ab196026, Abcam) for TPO-producing hepatocytes while spleen sections were stained with F4/80 AF647 (Host: Rat, Clone: BM8, Dilution: 1:200, Cat no: 123122, BioLegend), CD169 AF488 (Host: Rat, Clone: 3D6.112, Dilution: 1:200, Cat no: 142419, BioLegend) and unconjugated SIGNR1 (Host: Armenian hamster, Clone: eBio22D1 (22D1), Dilution: 1:50, Cat no: 14-2093-82, eBiosciences) for RP macrophages, marginal metallophilic macrophages (MMM) and marginal zone macrophages (MZM) respectively, for 45 minutes at RT. Slides were washed three times in wash buffer (3–5 minutes per wash with shaking). Liver sections were then incubated with goat anti-rabbit AF488 secondary antibody (Host: Goat, Clone: Polyclonal, Dilution: 1:200, Cat no: A-11034, Invitrogen) and spleen sections with goat anti-hamster AF546 secondary antibody (Host: Goat, Clone: Polyclonal, Dilution: 1:200, Cat no: A-21111, Invitrogen) for 30 minutes at RT. Slides were washed three times with wash buffer and twice with 1x PBS. All the tissue sections were counterstained with a nuclear stain, 4′,6-diamidino-2-phenylindole (DAPI; 1μg/ml in PBS) for 5 minutes. Slides were washed thoroughly with PBS and mounted with coverslips using ProLong® gold antifade mountant (Thermo Fisher Scientific, UK). Images were captured using AxioScan.Z1 Slide scanner (Zeiss, Oberkochen, Germany) at 20x resolution using Zen software (Zeiss, Oberkochen, Germany). Data were collected from 1–3 sections per mouse and pooled for analysis and processed for segmentation analysis using StrataQuest image analysis software (TissueGnostics, Vienna, Austria). Segmentation analysis was done by identifying cell nuclei and creating outlines around the cells based on the immunofluorescent staining. Cells with high auto-fluorescence were excluded from the final analysis. A similar strategy for segmentation analysis could be conducted in freely available open access software (e.g. Fiji ImageJ).

**Statistical analysis**

Data were analysed and figures constructed using GraphPad Prism 8 software (GraphPad Software, San Diego, CA). Alternative open access statistical analysis and graph making packages could be used (e.g. R). Parametric or non-parametric statistical tests were applied depending on the distribution of data and data represented as mean ± SD and median with quartiles respectively. Unpaired t test was used when comparing mean of two groups while ANOVA with post-hoc Tukey’s or Dennett’s and Kruskal Wallis with post-hoc Dunn’s tests were used when comparing more than two groups. A p-value of less than 0.05 was taken as significant, expressed as, * (< 0.05), ** (< 0.01), *** (< 0.001) and **** (< 0.0001).

## Results

### Restoration of hematological profile and tissue microarchitecture in AmBisome®-treated *L. donovani*-infected C57BL/6 mice

We have previously reported that seven days after single dose (8mg/kg) AmBisome® treatment, BALB/c mice show effective clearance of systemic parasite load and rapid resolution of the hepatic granulomatous response, but with an incomplete restoration of transcriptional homeostasis ^25^. We have also found that by four weeks after single dose (8mg/kg) AmBisome® treatment of C57BL/6 mice, there is complete restoration of platelet count, mean platelet volume (MPV), hepatomegaly, circulating and tissue TPO levels but only a partial restoration of splenomegaly ^26^. We therefore extended these key findings in the C57BL/6 model of VL to investigate other hematological and architectural changes post-treatment (Rx), using the same drug dosage and for a follow-up period of four weeks ( Figure 1A). In *L. donovani* infected C57BL/6 mice, restoration of blood parameters (Hb, hemoglobin; HCT, hematocrit; RBC, red blood cell count) had begun to occur by week one post-Rx and reached the normal range for all parameters measured by four weeks post-Rx ( Figure 1B–D) ^24^. Of note, red cell distribution width (RDW) increased during infection and continued to do so for two weeks post-Rx before it then returned to baseline levels ( Figure 1E), suggesting that erythrocytes of variable shapes and sizes are a feature of chronic *L. donovani* infection that recovers after parasite clearance. Changes to leucocytes were unremarkable during primary VL and post-Rx (extended data 1 ^24^).

**Figure 2.**
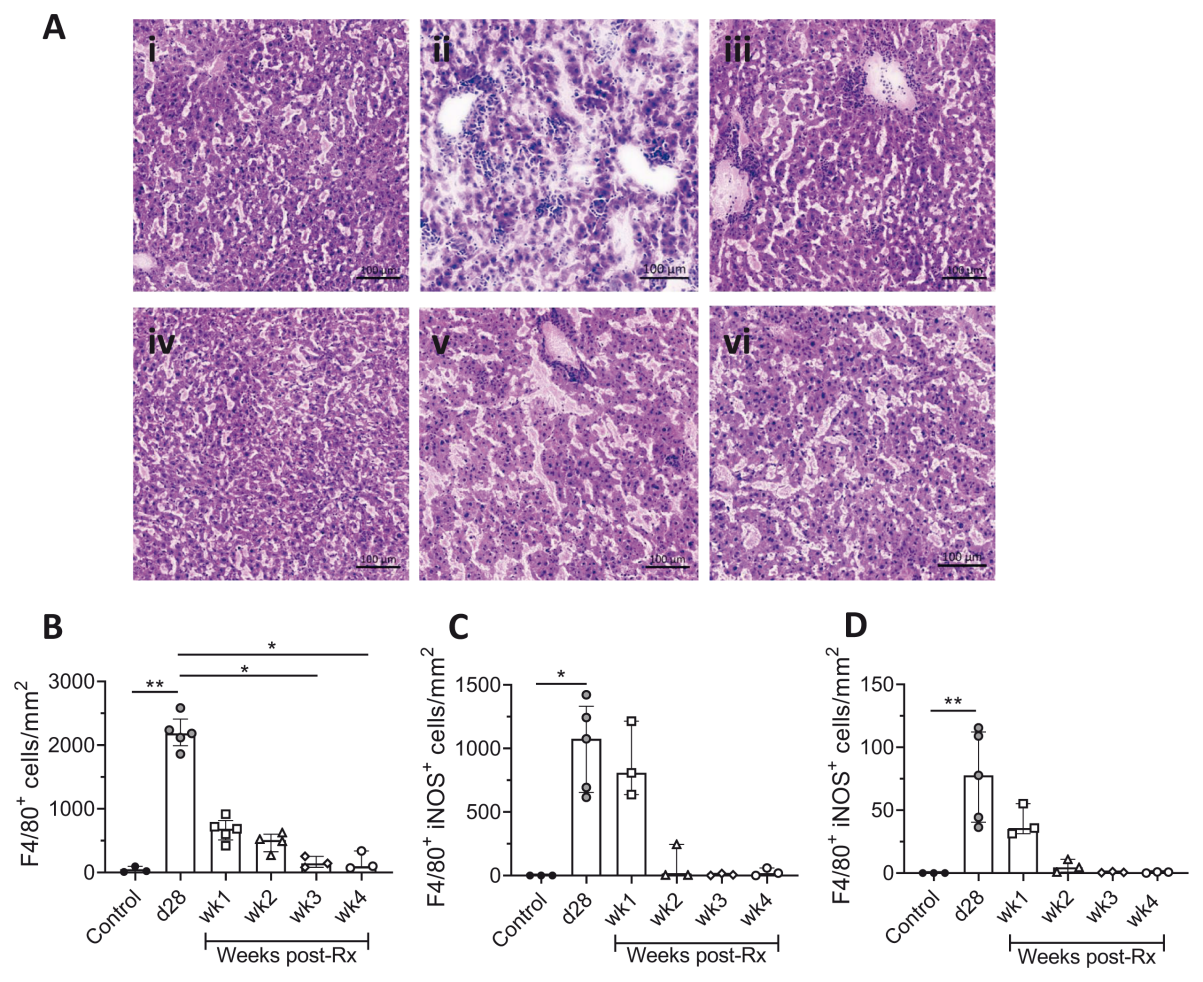
Restoration of hepatic microarchitecture in drug-treated *L. donovani* infected mice. **A**) H & E-stained liver sections of (i) uninfected control, (ii) d28, (iii) wk1, (iv) wk2, (v) wk3, (vi) wk4 post-Rx mice show the recovery of normal hepatic microarchitecture, scale bar; 100µm. **B**) Number of F4/80 ^+^ cells after treatment with AmBisome®, determined by segmentation analysis. Data are pooled from two independent experiments of 23 mice, with n=3 uninfected controls, n=5 d28 infected and n=3-5 treated mice per time-point. Data are expressed as median with quartiles and analysed using non-parametric Kruskal Wallis with Dunn’s post-hoc test, *, p < 0.05; **, p < 0.01. **C** and **D** Number of iNOS ^+^ F4/80 ^+^ cells per unit area inside ( **C**) and outside ( **D**) hepatic granulomas, determined by segmentation analysis in StrataQuest. Data are derived from n=3 uninfected control mice, n=5 d28 infected mice and n=3 treated mice at weeks 1–4. Data shown as median with quartiles and were analysed using non-parametric Kruskal-Wallis test with Dunn’s post-hoc test, *, p < 0.05; **, p < 0.01.

No parasites were found on the Giemsa-stained tissue impression smears over the four-week follow-up, in either spleen or liver, and both tissues displayed signs of restoration of homeostasis. Hematoxylin and eosin (H&E) staining of liver sections confirmed these findings with a reduction in the sizes of hepatic granulomas and restoration of normal liver architecture ( Figure 2A). A decrease in the number of F4/80 ^+^ macrophages provided additional evidence of architectural restoration in the liver ( Figure 2B). Furthermore, a reduction in the number of F4/80 ^+^iNOS ^+^ cells, both inside and outside the resolving hepatic granulomas was observed ( Figure 2C–D), suggesting a decline in hepatic macrophage activation status.

**Figure 3.**
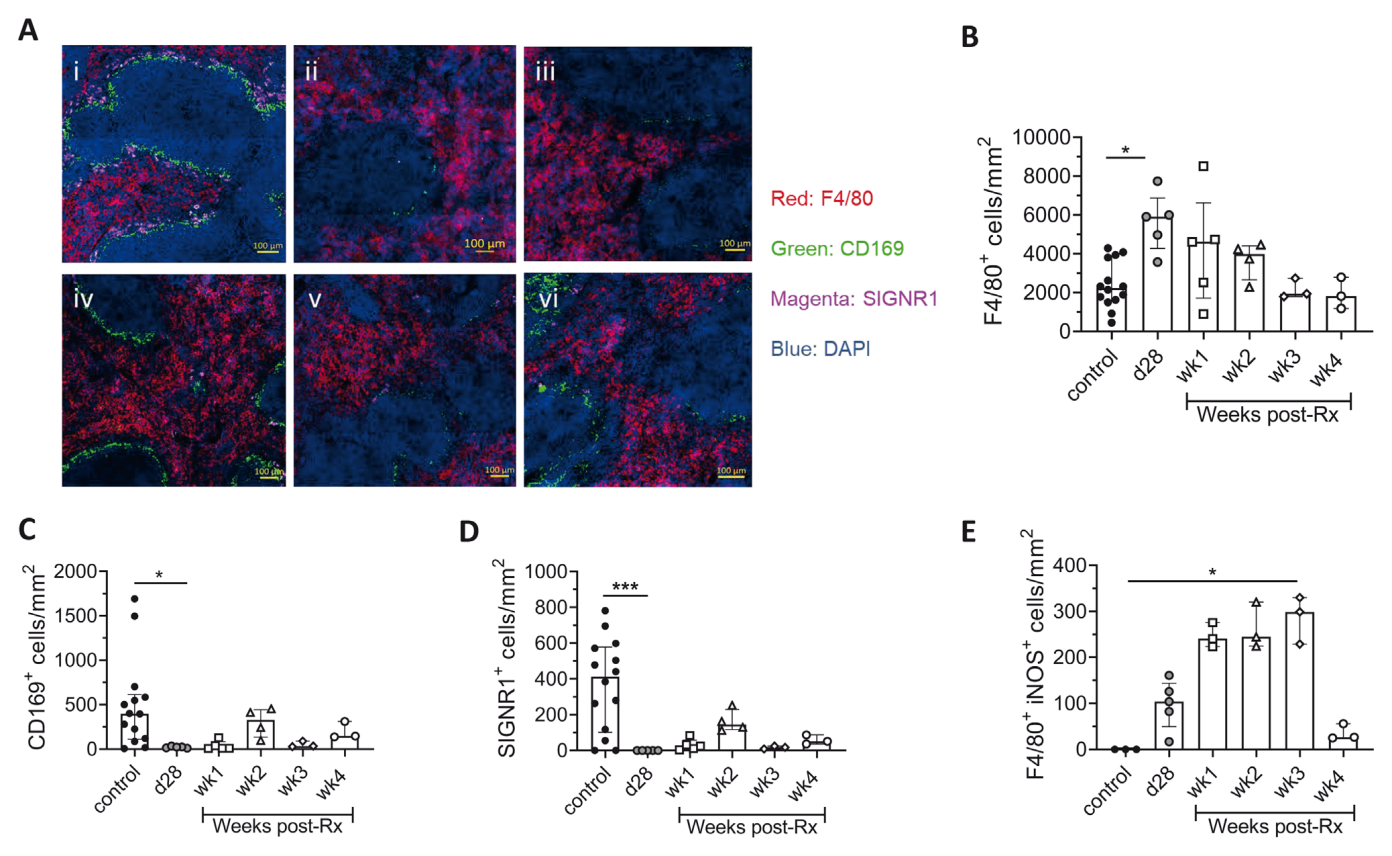
Restoration of splenic microarchitecture in drug-treated *L. donovani* infected mice. **A**) Spleen sections of (i) uninfected control, (ii) d28, (iii) wk1, (iv) wk2, (v) wk3, (vi) wk4. post-Rx mice were stained with macrophage markers, DAPI (Blue); nuclei, F4/80 (Red); RP macrophages, CD169 (Green) and SIGNR1 (Magenta) for MMM and MZM respectively, scale bar; 100µm. **B**– **D**) The number of F4/80 ^+^ RP macrophages ( **B**), CD169 ^+^ MMM ( **C**) and SIGNR1 ^+^ MZM ( **D**) after treatment with AmBisome® was determined by segmentation analysis. Data are pooled from two independent experiments with n=14 uninfected control, n=5 d28 infected and n=3-5 treated mice per time-point post-Rx. Data shown as median with quartiles and were analysed using non-parametric Kruskal-Wallis test with Dunn’s post-hoc test, *, p < 0.05; ***, p < 0.001. **E**) The number of iNOS ^+^ F4/80 ^+^ cells per unit area was determined by segmentation analysis. Data are representative of n=3 uninfected control mice, n=5 d28 infected mice and n=3 treated mice at weeks 1–4 post-Rx. Data shown as median with quartiles and were analysed using non-parametric Kruskal-Wallis test with Dunn’s post-hoc test, *, p < 0.05.

A partial recovery of macrophages in the splenic marginal zone, including marginal zone macrophages (MZM) and marginal metallophilic macrophages (MMM) and a reduction in red pulp (RP) macrophages was also indicative of some restoration of splenic microarchitecture post-Rx ( Figure 3A). A progressive reduction in F4/80 ^+^ RP macrophages and trend towards slow restoration of CD169 ^+^ MMM and SIGNR1 ^+^ MZM was seen over a period of four weeks post-Rx ( Figure 3B–D). A trend towards an increase in the number of iNOS ^+^ splenic RP macrophages was also observed for the initial three weeks post-Rx followed by a sharp decline at four weeks post-Rx ( Figure 3E), suggestive of a transient increase in splenic macrophage activation associated with parasite death in this organ, with some residual activation remaining at four weeks post Rx.

In summary, four weeks after a curative dose of AmBisome®, C57BL/6 mice recovered from many of the salient features of primary experimental VL, but as with humans discharged following treatment, showed some signs of persistent splenic pathology. No unexpected adverse effects were observed in either experimental or control groups throughout the course of experiment.

### Clinical outcome of sequential *P. chabaudi* infection in mice previously infected and cured from *L. donovani* infection

**Figure 4.**
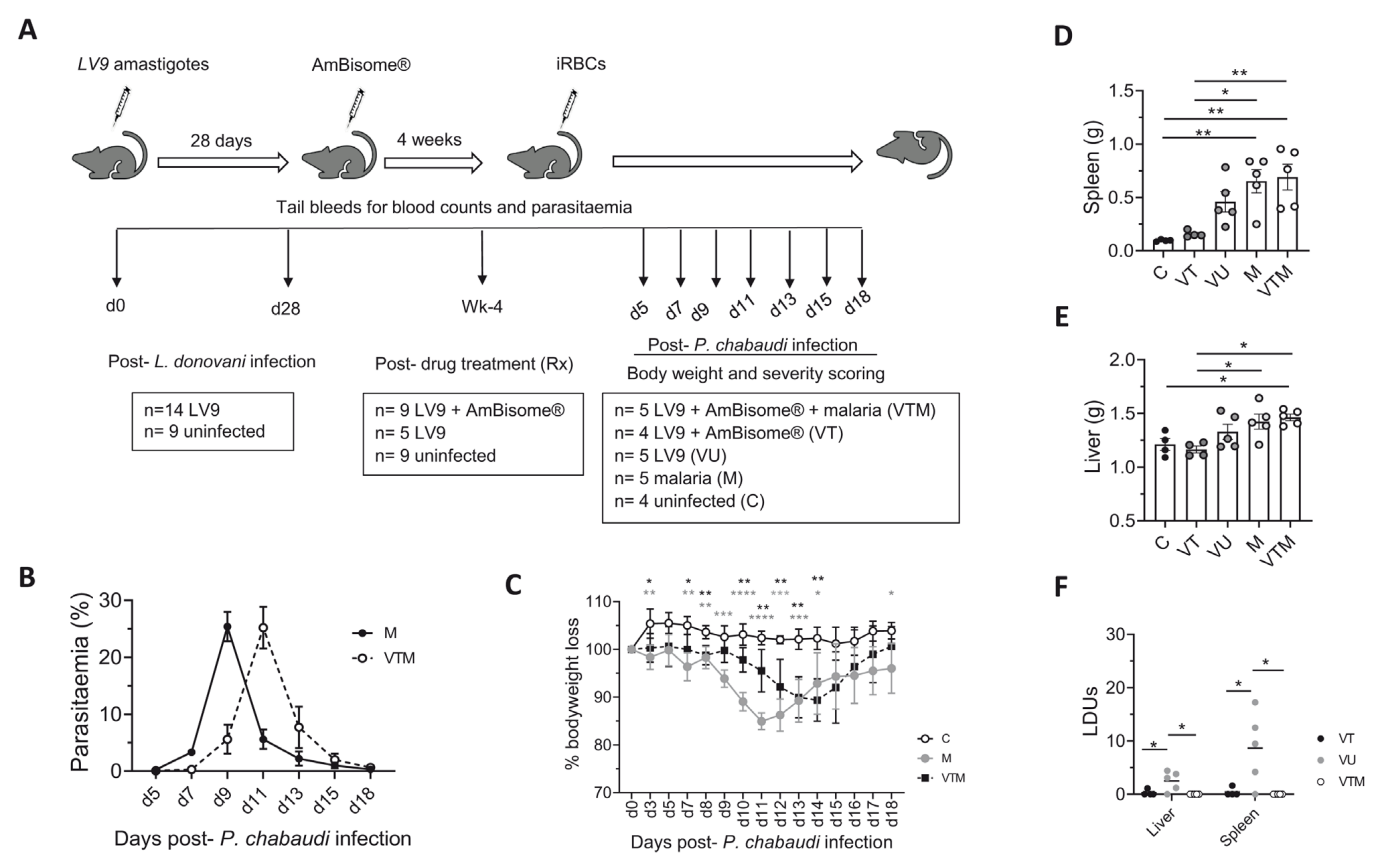
Disease kinetics of malaria infection in drug-treated *L. donovani* infected mice. **A**) A cohort of 14 *L. donovani*-infected C57BL/6 mice were randomly allocated to receive single dose of AmBisome® (8mg/kg) at d28 p.i (n=9) or no treatment (VU; n=5). Four weeks post-Rx, AmBisome®-treated mice were randomly allocated to receive 1 × 10 ^5^ *P. c. chabaudi (AS)* iRBCs intravenously (VTM; n=5) or no malaria infection (VT; n=4). Control mice included mice infected only with *P. c. chabaudi (AS)* (M; n=5), *L. donovani*-infected but untreated mice (VU; n=5), and control naïve mice (C; n=4). Mice were kept under strict 12-hour light-dark cycle and monitored regularly for parasitemia, weight loss, blood counts and signs of disease severity. **B**) Parasitemia in malaria-infected mice (M, VTM) determined from Giemsa-stained blood smears. Data is shown as % body weight loss compared to day 0. **C**) % bodyweight loss was determined in control (C) and malaria-infected (M, VTM) mice at the times indicated. Data were analysed using ANOVA with Dennett’s post-hoc test comparing mean ± SD of M and VTM groups with C group, *, p < 0.05; **, p < 0.01; ***, p < 0.001; ****, p < 0.0001. **D** and **E**). Post-mortem spleen ( **D**) and liver ( **E**) weights were determined in control ( **C**, VT, VU) and malaria-infected (M, VTM) groups. Data were analysed using ANOVA with Tukey’s post-hoc test, *, p < 0.05; **, p < 0.01. ( **F**) *Leishmania* parasite burden in spleen and liver was determined from Giemsa-stained tissue impression smears of tissues from VT, VU and VTM mice. Data are shown as LDU. Data were analysed using ANOVA with Tukey’s post-hoc test, *, p < 0.05.

To determine whether previous *L. donovani* infection and AmBisome® treatment altered the outcome of primary *P. chabaudi* infection, we compared sequentially infected mice (VTM) with mice receiving only primary infection with *P. chabaudi* (M) as indicated in Figure 4A. In addition to naïve control mice (C), additional control groups of mice included: i) mice infected and cured of *L. donovani* infection but without malaria infection (VT) to ascertain whether spontaneous VL relapse occurred, and ii) mice infected with *L. donovani* but untreated (VU) to monitor natural progression of the primary infection ( Figure 4A).

Parasitemia was determined over 18 days in all *P. chabaudi*-infected mice (M, VTM). Although the peak *P. chabaudi* parasitemia was equivalent in M and VTM mice, time to peak parasitemia was delayed by two days in VTM mice ( Figure 4B). This delay in parasitemia was mirrored in a delayed loss of body weight in VTM compared to M mice ( Figure 4C). At day 18 p.i, *P. chabaudi* splenomegaly was similar between M and VTM mice ( Figure 4D) but hepatomegaly was minimal in both groups ( Figure 4E). We also determined whether sequential *P. chabaudi* infection led to a relapse of primary VL, by comparing *L. donovani* tissue parasite load in VU, VT and VTM mice ( Figure 4F). No differences were observed in *L. donovani* load between VT and VTM mice, indicating that subsequent malaria infection did not trigger relapse of *L. donovani* infection. In comparison to VT mice, small numbers of *Leishmania* amastigotes were detectable in VU mice, likely reflecting a slow natural recrudescence of parasites in C57BL/6 mice by seven weeks after AmBisome® treatment. Thus, under these experimental conditions, sequential *P. chabaudi* infection did not lead to increased tissue amastigote burden. No unexpected adverse effects were observed in either experimental or control groups throughout the course of experiment.

**Figure 5.**
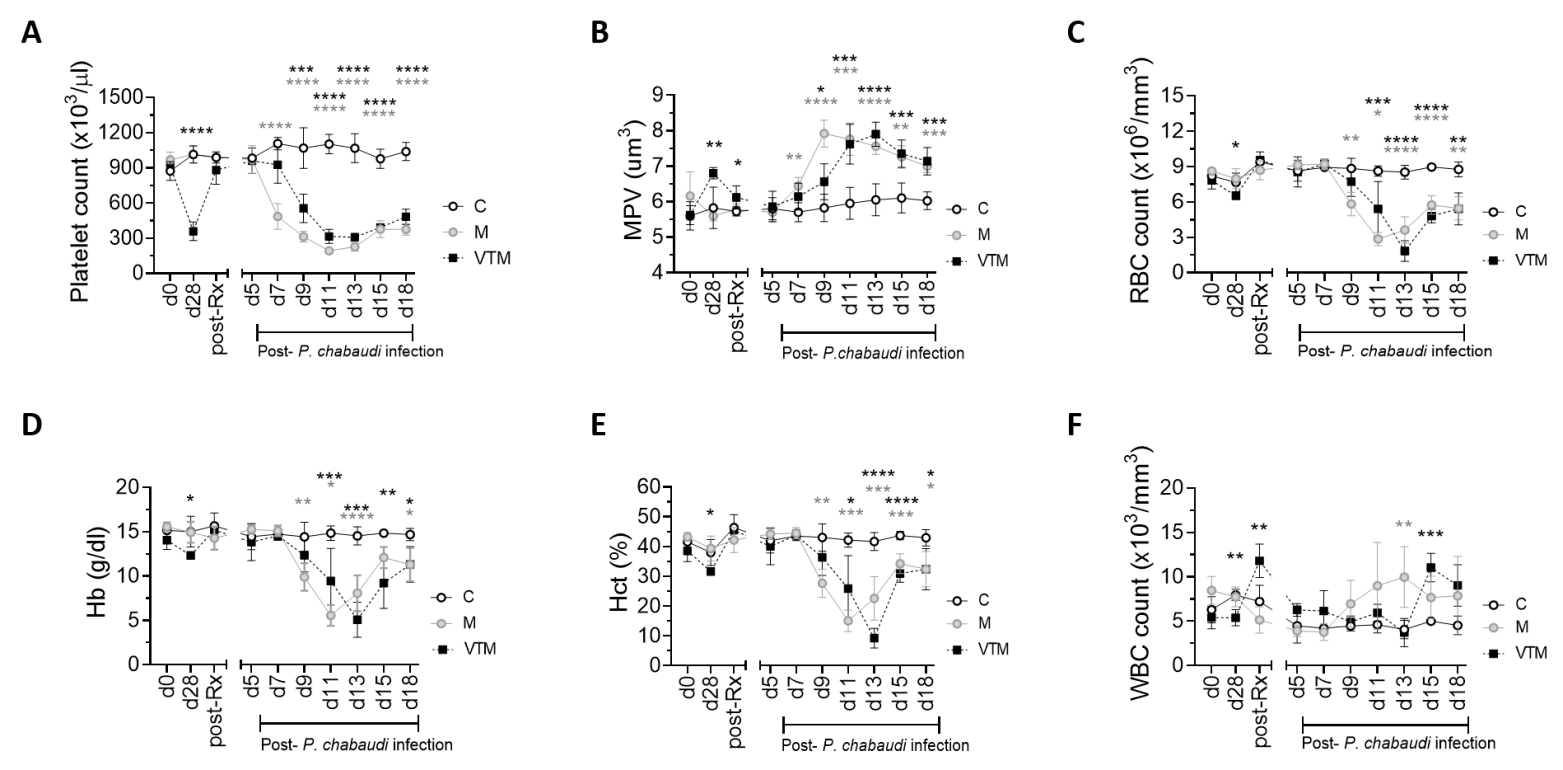
Hematological changes in response to malaria infection in drug-treated *L. donovani* infected mice. **A**– **E**) Platelet count ( **A**) mean platelet volume (MPV; **B**) total RBC count ( **C**) hemoglobin (Hb; **D**), hematocrit (Hct; **E**) and total white blood cell count. (WBC; **F**) were determined in *P. c. chabaudi (AS)* infected mice (M), *P. c. chabaudi (AS)* infected mice with previously-treated *L. donovani* infection (VTM) and control mice (C) were determined at the times indicated. All data are representative of a single experiment with n=4 C, n=4 VT, n=5 VU, n=5 M and n=5 VTM mice. Data were analysed using ANOVA with Dennett’s post-hoc test comparing mean ± SD of M and VTM groups with C group,*, p < 0.05; **, p < 0.01; ***, p < 0.001; ****, p < 0.0001.

In VTM mice, we observed a delayed hematological response to malaria as compared to M mice, consistent with the delayed kinetics of the *P. chabaudi* infection. As anticipated ^26^, we observed in VTM mice that the thrombocytopenia associated with primary *L. donovani* infection was fully reversed by four weeks post AmBisome® treatment. Delayed onset of thrombocytopenia after *P. chabaudi* infection and early recovery of platelet count was observed in these mice as compared to M mice ( Figure 5A), though the rate of thrombocytopenia progression and severity were similar in both groups. The increase in platelet volume (MPV) was also delayed in VTM mice as compared to M ( Figure 5B). Similarly, malaria-induced anemia was delayed in the VTM group in comparison to M group, assessed by RBC count, Hb and Hct ( Figure 5C–E). Other red cell indices MCV, MCH, MCHC and RDW showed a similar pattern in both groups.

 Leucopenia and leucocytosis both are reported as a feature of *Plasmodium* infection ^27, 28^ and total WBC count began to rise with increasing parasitemia in M group mice and at a later time in VTM mice ( Figure 5F). Our data show an increase in total WBC count after d13 of malaria when parasitemia started to decline ( Figure 5F). Together these data suggest that hematological changes in the peripheral blood are sensitive to the delayed rise in parasitemia observed in VTM mice. No significant difference in any of the blood parameters was observed in control groups (C, VT). A slow recovery of blood counts was seen in VU mice suggestive of natural self-resolution of infection in mice (extended data 2 ^24^ and 29).

**Figure 6.**
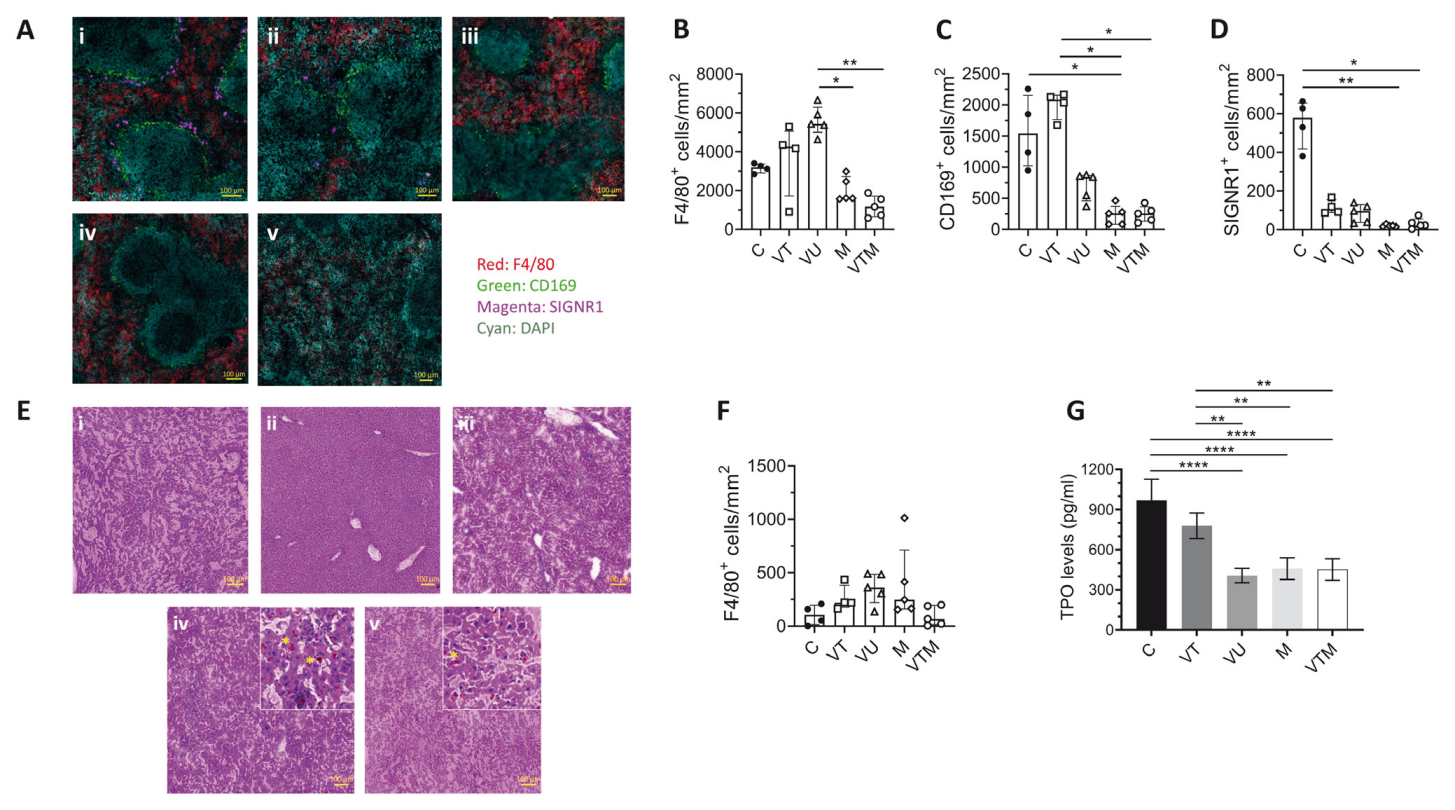
Tissue microarchitectural changes in response to malaria infection in drug-treated *L. donovani* infected mice. **A**) Spleen sections of (i) C, (ii) VT, (iii) VU, (iv) M and (v) VTM mice killed 18 days post *P. c. chabaudi (AS)* infection (see Figure 4A) were stained with macrophage markers, F4/80 (Red); RP macrophages, CD169 (Green) and SIGNR1 (Magenta). Scale bar; 100µm. **B**– **D)** Number of F4/80 ^+^ RP macrophages ( **B**), CD169 ^+^ MMM ( **C**) and SIGNR1 ^+^ MZM ( **D**) in indicated groups. Data shown as median with quartiles and were analysed using non-parametric Kruskal-Wallis with post-hoc Dunn’s test comparing the median of each group, *, p < 0.05; **, p < 0.01. **E**) H & E-stained liver images of (i) C, (ii) VT, (iii) VU, (iv) M and (v) VTM mice. Orange-red pigment is hemozoin (*). **F**) Number of F4/80 ^+^ cells per unit area, determined by segmentation analysis for each mouse group. Data shown as median with quartiles of n=4 C, n=4 VT, n=5 VU, n=5 M and n=5 VTM mice. **G**) Circulating thrombopoietin (TPO) was determined in serum samples of each mouse group by ELISA at the end of the experiment (day 18 post *P. c. chabaudi (AS)* infection). Data show the results of a single experiment with n=4 C, n=4 VT, n=3 VU, n=4 M and n=4 VTM mice. Data shown as mean ± SD and were analysed using ANOVA with Tukey’s as post-hoc test, **, p < 0.01; ****, p < 0.0001.

Quantitative immunohistochemistry analysis, on mice killed 18 days after *P. chabaudi* infection, was used to examine whether there were any synergistic or antagonistic effects on splenic architecture between single and sequentially infected mice ( Figure 6A). M and VTM mice had similarly reduced numbers of F4/80 ^+^ RP macrophages per unit area compared to VU mice ( Figure 6B), not dissimilar to C and VT mice. Additionally, the destruction of the splenic marginal zone was also similar in malaria-infected mice. As expected, recovery of CD169 ^+ ^MMM appeared to be greater in VT mice at seven weeks post AmBisome® treatment ( Figure 6C) compared to at four weeks post treatment ( Figure 3C). In contrast, complete restoration of SIGNR1 ^+ ^MZM was not observed even at this later time point in VT spleens, and a further reduction was noticed in malaria-infected mice ( Figure 6D), suggestive of cumulative pathological remodeling.

The architectural changes in the livers of malaria-infected mice were consistent with the published literature including the presence of orange-red malaria pigment, hemozoin ( Figure 6E). The number of F4/80 ^+^ cells were not significantly different across treatment groups and controls ( Figure 6F). Serum TPO levels were also analysed in all groups of mice, as this has previously been correlated with thrombocytopenia. VT mice had similar serum TPO concentration to C mice, indicative of treatment response and significant restoration of blood homeostasis ( Figure 6G). In contrast, VU, VTM and M mice all displayed reduced serum TPO levels, approximating 50% of that seen in C mice ( Figure 6G), consistent with the similar levels of thrombocytopenia observed in these mice ( Figure 5A and Extended Data 2). Hence, both *L. donovani* and *P. chabaudi* infections reduce TPO levels to a similar extent with no indication of synergistic or antagonistic activity.

## Discussion

Both leishmaniasis and malaria are among the tropical diseases with a huge overlap of geographical and clinical presentation ^11, 30– 33^. The presence of coinfections with VL not only makes it difficult to treat but may also pose a risk of relapse in treated cases ^34^. Although VL/HIV coinfection is prevalent and well-studied, there is an increasing incidence of other infections coexisting with VL ^11, 13, 32, 33, 35^. In this study, we established a model of a sequential *P. chabaudi* infection in a AmBisome®-treated murine VL model to specifically address the question of whether mice previously exposed to *L. donovani* and cured using a first line therapy would differ in their subsequent response to malaria challenge. 

As previously documented ^25^, *L. donovani*-infected BALB/c mice treated with a single dose of AmBisome® (8mg/kg) at d28 p.i. showed a progressive recovery towards immune homeostasis. Here, we extend our previous study by showing that the resolution of hepatic granulomatous inflammation, imputed from transcriptomic analysis and quantified in H&E-stained liver sections, is also accompanied by a reduction in the activation status of F4/80 ^+^ hepatic macrophages (predominantly Kupffer cells), measured by iNOS expression. In the spleen, accompanying the reduction in splenomegaly previously reported ^25^, we now formally document the partial recovery of populations of marginal zone macrophages and provide evidence for the activation status of splenic macrophages. As expected, based on early studies of repopulation kinetics following clodronate depletion ^36^, recovery of MZMs and MMMs was slow and incomplete at four weeks post treatment. One week after AmBisome® treatment, the abundance of red pulp F4/80 ^+^ iNOS ^+^ macrophages increased significantly. This was in contrast to our previous transcriptomic data that indicated a reduction in overall tissue *Nos2* accumulation at this time point ^25^, suggesting that other cell populations also play an important role in iNOS production during active VL. The number of F4/80 ^+^ iNOS ^+^ cells was maintained over the following two weeks before declining at four weeks to a level that remained above that seen in naïve control mice. This analysis is consistent with a residual level of macrophage activation in the red pulp at the time of malaria challenge in this study and with a previous report which found that activated red pulp macrophages during *L. donovani* infection can have enhanced phagocytic capacity towards heterologous pathogens ^6^.

We used these *L. donovani*-infected, drug treated mice to determine whether there was any impact on the subsequent development of a primary *P. chabaudi* infection. Whilst there was a clear delay in the kinetics of all parasitological and clinical parameters measured, including parasitemia, loss of body weight and cytopenia, there was no quantitative differences in the severity of malaria between sequentially infected mice (VTM) and control mice infected only with *P. chabaudi* (M). Histological changes in the liver and spleen of VTM mice were consistent with that expected from a single *P. chabaudi* infection (this report and 37).

Recent attention has been focused on the ability of previous exposure to train the innate immune system for heightened responsiveness on secondary heterologous challenge, so called trained immunity ^38, 39^. Trained immunity has been most well-studied in phagocytes exposed to strong agonists of innate pattern recognition receptors such as BCG or β-glucan, and may involve both epigenetic and metabolic reprogramming in the periphery or at the level of bone marrow stem cells ^40, 41^. Although we have not formally addressed whether *L. donovani* infection can stimulate trained immunity, given our current data a likely explanation for the delay in malaria kinetics is that F4/80 ^+^ iNOS ^+ ^cells in the spleen have enhanced capacity to clear iRBCs ^42^ and hence the initial parasite load establishing the malaria infection is reduced. Further studies would be required to test this hypothesis formally and/ or to establish a role for trained immunity following *L. donovani* infection.

A weakness of the current study is that malaria infection was initiated by needle challenge using iRBCs, rather than via mosquito bite ^43, 44^. Hence, it is not possible with this model to ascertain whether changes to the liver microenvironment that remain after cure from VL might impact on the establishment of the exoerythrocytic stages of the malaria life cycle. Whilst we did not see residual iNOS activity in the liver and hepatic granulomatous inflammation had subsided significantly one month after treatment (this report and ref 25), more subtle changes to hepatic endothelial cells, Kupffer cells or hepatocytes that affect sporozoite invasion and / or exo-erythrocytic schizogony cannot be excluded.

In humans, trials of short course AmBisome® treatment in Bangladesh have indicated that residual splenomegaly one month after the initiation of treatment is a risk factor for relapse ^45^, mirroring earlier data from patients treated with AmBisome® in India ^14^ and with sodium stibogluconate and paromomycin in South Sudan ^15^. Hence, it seemed reasonable in the absence of other data to evaluate responses to malaria infection one month after AmBisome® treatment in this murine model. We cannot exclude the possibility that alternate schedules of sequential infection may lead to differing outcomes. Similarly, the mouse model of VL fails to display the full severity and duration of human VL, both factors that might influence bone marrow function with respect to platelet and erythrocyte production as well as other parameters of immunity to malaria. Studies in the more severe hamster model of VL may be warranted. Whilst a previous study examined coinfection with the cutaneous parasite *Leishmania enriettii* and *P. berghei* ^46^, malaria infections in the hamster are relatively poorly understood, potentially limiting the value of such a model.

In summary, our data indicate that despite similar impacts on peripheral blood red cell and platelet counts, and evidence for hematological disturbances associated with both malaria and visceral leishmaniasis, we found that sequential infection led only to a delay in primary malaria parasitemia, with minimal impact on other clinical or histopathological features. Although studies in animal models such as the one described have value for mechanistic studies, given the limitations imposed by these models, the question of how infections interact in a sequential manner should be studied in parallel through longitudinal population-based studies in humans naturally exposed to both pathogens.

## Data availability

### Underlying data

Open Science Framework: Impact of prior visceral leishmaniasis on subsequent malaria infection in mice. https://doi.org/10.17605/OSF.IO/DSVCP ^24^

This project contains the following underlying data:

- Sample size calculations Raw data
- Figure 1 Raw data (Schem Recovery of red blood cell parameters in drug-treated *L. donovani* infected mice.)
- Figure 2 Raw data (Restoration of hepatic microarchitecture in drug-treated *L. donovani* infected mice.)
- Figure 3 Raw data Restoration of splenic microarchitecture in drug-treated *L. donovani* infected mice.
- Figure 4 Raw data (Disease kinetics of malaria infection in drug-treated *L. donovani* infected mice).
- Figure 5 Raw data (Hematological changes in response to malaria infection in drug-treated *L. donovani* infected mice).
- Figure 6 Raw data (Tissue microarchitectural changes in response to malaria infection in drug-treated *L. donovani* infected mice).
- Extended data 1 Raw Data (Leucocyte counts in drug-treated *L. donovani* infected mice)
- Extended data 2 Raw data (Hematological changes in the control groups of drug-treated *L. donovani* infected mice)

### Extended data

Open Science Framework: Impact of prior visceral leishmaniasis on subsequent malaria infection in mice. https://doi.org/10.17605/OSF.IO/DSVCP ^24^

This project contains the following extended data:

- Extended data 1 (Leucocyte counts in drug-treated *L. donovani* infected mice)
- Extended data 2 (Hematological changes in the control groups of drug-treated *L. donovani* infected mice)

Data are available under the terms of the Creative Commons Attribution-ShareAlike 4.0 International (CC BY-SA 4.0).
