# Supplementary material for: Hematological consequences of malaria in mice previously treated for visceral leishmaniasis
Source: Wellcome Open Res. 2021 Jun 18;6:83. Originally published 2021 Apr 16. [Version 2] doi: 10.12688/wellcomeopenres.16629.2 (PMC8276186; doi:10.12688/wellcomeopenres.16629.2)
Supplement: Peer review history

# Peer review: Reviewer response for version 2

**DOI:** 10.21956/wellcomeopenres.18750.r44578

**Published:** 2021-07-09

**Authors:** Yasuyuki Goto

**Affiliations:** 1 Laboratory of Molecular Immunology, Graduate School of Agricultural and Life Sciences, The University of Tokyo, Tokyo, Japan

The authors have well responded to my comments and made enough modifications.

Is the work clearly and accurately presented and does it cite the current literature?

Yes

If applicable, is the statistical analysis and its interpretation appropriate?

I cannot comment. A qualified statistician is required.

Are all the source data underlying the results available to ensure full reproducibility?

Partly

Is the study design appropriate and is the work technically sound?

Yes

Are the conclusions drawn adequately supported by the results?

Yes

Are sufficient details of methods and analysis provided to allow replication by others?

Yes

Reviewer Expertise:

leishmaniasis

I confirm that I have read this submission and believe that I have an appropriate level of expertise to confirm that it is of an acceptable scientific standard.

# Peer review: Reviewer response for version 1

**DOI:** 10.21956/wellcomeopenres.18333.r43545

**Published:** 2021-05-05

**Authors:** Breanna M. Scorza

**Affiliations:** 1 Department of Epidemiology, University of Iowa, Iowa City, IA, USA

**Summary:**

 Polyparasitism between Visceral Leishmaniasis (VL) and Malaria is well documented and occurs at varying rates across endemic areas. The authors note that studying diseases in the context of how they might occur in naturally affected populations is critical to translational research and often experimental models are overly simplistic compared to the natural infection setting. For example, recent research has shown that previous pathogen experience can alter immune responses to subsequent heterologous infections by modulating the microenvironment. Here the authors establish a mouse model system to test the effect of previous VL and treatment on subsequent Malaria disease.

 VL is established with a high dose of i.v. parasites, allowed to progress for 28 days, followed by treatment with AmbiSome, a frontline VL drug. The VL disease measurements have almost entirely returned to baseline 4wks post treatment. Four weeks post treatment, Malaria is initiated through iRBCs with rodent adapted plasmodium. Plasmodium parasitemia and disease severity are slightly delayed in the treated VL mice, however, no change increasing or decreasing response is observed. A weakness of the model is initiating malaria using blood stage parasites and bypassing the liver stage of infection, which may very well be affected by previous VL at this bottleneck. The authors conclude that VL in this model system did not change the immune environment sufficiently to affect malaria outcomes however, they acknowledge that their system is again perhaps too simplistic. Mice do not experience full VL disease compared to humans, dogs, or hamsters. They suggest longitudinal human studies in endemic areas as the best system to assess this complex pathogen ecology question.

 The experimental model is well designed and presented. The system could use more technical replicates; however, the results seem to be quite clear even with the small number of mice at the later timepoints. The parameters used to assess VL or malaria disease are appropriate and give a good clinical picture. This study will be important for researchers studying coinfections or interactions between these parasitic diseases and should be published so as not to be repeated exactly. I agree with the authors that the findings presented here may well be different in a human study, where more chronic VL usually occurs and more long-lasting changes to the splenic architecture and bone marrow compartment may be experienced leading to different outcomes on subsequent infection. Human VL is also usually associated with malnutrition and lower socioeconomic standing, which could affect the immune microenvironment in itself.

**Minor comments:**

 The authors did not address any adaptive immune functions in this model. Based off the clinical outcomes not significantly varying, I would assume the malaria adaptive response developed normally however one of the tenets underlying the reasoning for performing this study is that downstream effects may occur on subsequent infections. I wonder if the authors could speculate on whether the formation of immune memory or a secondary Malaria challenge would look like.

 In the immunofluorescence images, the magenta SIGNR1 signal is very hard to discern apart from the red F4/80. In the future, white, yellow, or another color should be used to delineate these markers. Since the quantification is present, it does not prevent the reader from interpreting the results.

Is the work clearly and accurately presented and does it cite the current literature?

Yes

If applicable, is the statistical analysis and its interpretation appropriate?

Yes

Are all the source data underlying the results available to ensure full reproducibility?

Yes

Is the study design appropriate and is the work technically sound?

Yes

Are the conclusions drawn adequately supported by the results?

Yes

Are sufficient details of methods and analysis provided to allow replication by others?

Yes

Reviewer Expertise:

Leishmaniasis, immunology, host pathogen interactions, parasitology

I confirm that I have read this submission and believe that I have an appropriate level of expertise to confirm that it is of an acceptable scientific standard.

# Response

**Published:** 2021-06-08

**Authors:** Paul Kaye

**Affiliations:** Department of Biology and Hull York Medical School, Hull York Medical School, University of York, UK

We thank the reviewer for her supportive comments.  We did not measure acquired immune responses to Plasmodium infection, but as the reviewer points out, we would not expect there to be major changes besides perhaps a delay in kinetics.  We would prefer not to speculate on whether previous VL might impact memory generated during primary *Plasmodium *infection, but in our revised manuscript we have noted this as a possible area for further study (Discussion, paragraph 5) .

# Peer review: Reviewer response for version 1

**DOI:** 10.21956/wellcomeopenres.18333.r43552

**Published:** 2021-05-04

**Authors:** Yasuyuki Goto

**Affiliations:** 1 Laboratory of Molecular Immunology, Graduate School of Agricultural and Life Sciences, The University of Tokyo, Tokyo, Japan

Due to a huge geographical overlap of VL and malaria, it is important to study how infection of one parasite affects infection with the other. Rani *et al.* further the topic by addressing the influence of past chemotherapy for VL to the subsequent *P. chabaudi *infection. Though a difference in the day of peak parasitemia was observed between VL-naïve and VL-treated mice upon *P. chabaudi *infection, no other major differences were found, suggesting that recovery from VL has limited impact on malaria pathology.

 The paper is well written and the data shown here are sufficient to draw the presented conclusions. Additionally, discussions on the limitations of the study, including the infection route of *P. chabaudi*, un-sustained pathology of VL in the used mouse model and only one time point of post-treatment tested for influence to the subsequent infection, are fair.

 However, the motivation for studying the influence of recovery from VL to malaria is still weak, because it is not clear what situation, in reality, the authors tried to simulate by this model. The timing of Plasmodium infection was 4 weeks after AmBisome treatment when splenomegaly still remained. This means the model would not reflect what happens in malaria on the majority of people with VL history. Though the authors discussed the limitation of testing only one post-treatment time point in this study, more careful discussions on what this model would reflect in a real situation are encouraged. The authors mentioned residual splenomegaly after treatment as a risk factor, which is just an indicator of incomplete treatment of VL which brings a higher chance of VL relapse and is irrelevant to the study objective which addresses the influence on other infections. Besides discussions on trained immunity, any chances for T/B cell cross reactivity between Leishmania and Plasmodium? This kind of discussion would be helpful to deliver the significance of why influence between Leishmania and Plasmodium was studied at the selected time point.

 Please describe the total number of repeated experiments in M&M.

 Figure 2B and elsewhere: Some data are pooled from two independent experiments, but why? The number of animals per group does not seem increased (n=3 for uninfected controls) even by pooling.

 Figure 6E: Why (i) C and (ii) VT look so different? They should look similar according to Figure 2. Besides, the resolution of the images is low. Images with better quality should be posted as extended data if there is a space limit in the main text.

 Other minor comments:

- Page 3 and elsewhere: Infection with malaria/malaria infection is better modified to infection with *Plasmodium/Plasmodium *infection.
- Page 3 and elsewhere: p.i to p.i.
- Page 3: About the sentence of ‘Mice were kept...’ in the paragraph of malaria infection on Procedures of M&M, I believe the 12-hour cycle is applied to all the mice even those without infection, correct? If different, please describe the light/dark cycle taken for non-infected mice.
- Page 5: No need to repeat the detail on F4/80 AF647 in M&M.
- Page 5: DAPI; 1μg/ml: need space between the number and the unit.
- Page 8: ‘In comparison to VT mice, ...’ This does not make sense. VU should be ‘untreated’.
- Page 9/Figure 5A: ‘Delayed onset of thrombocytopenia after *P. chabaudi *infection and early recovery of platelet count…’ Not sure if the data show ‘early recovery’.
- Figure 1: i.v to i.v.
- Figure 4C: Not % BW ‘loss’, just % BW.
- Figure 4F: Are LDUs of VU at this time point supposed to be that low in both the spleen and liver?

Is the work clearly and accurately presented and does it cite the current literature?

Yes

If applicable, is the statistical analysis and its interpretation appropriate?

I cannot comment. A qualified statistician is required.

Are all the source data underlying the results available to ensure full reproducibility?

Partly

Is the study design appropriate and is the work technically sound?

Yes

Are the conclusions drawn adequately supported by the results?

Yes

Are sufficient details of methods and analysis provided to allow replication by others?

Yes

Reviewer Expertise:

leishmaniasis

I confirm that I have read this submission and believe that I have an appropriate level of expertise to confirm that it is of an acceptable scientific standard, however I have significant reservations, as outlined above.

# Response

**Published:** 2021-06-14

**Authors:** Paul Kaye

**Affiliations:** Department of Biology and Hull York Medical School, Hull York Medical School, University of York, UK

We thank the reviewer for his detailed critique and have improved the manuscript by addressing these as follows:

*…..This means the model would not reflect what happens in malaria on the majority of people with VL history…….The authors mentioned residual splenomegaly after treatment as a risk factor, which is just an indicator of incomplete treatment of VL which brings a higher chance of VL relapse and is irrelevant to the study objective which addresses the influence on other infections.*

 We did not intend to imply that there was evidence that residual splenomegaly was a risk factor for malaria, as this was the hypothesis under test in this study.  We have now clarified this point (new text: Introduction paragraph 2).   We agree with the reviewer that a majority of people with malaria following VL treatment are likely to have acquired their malaria at later times post treatment than modelled here.  However, in areas of high malaria endemicity, exposure to *Plasmodium *could readily occur post discharge and before a return to full immune and / or hematological homeostasis.  Hence, we believe that this model does reflect possible patient experience.  However, we have amended the discussion to reflect the reviewer’s comments (new text: Discussion paragraph 6).    

*……any chances for T/B cell cross reactivity between Leishmania and Plasmodium? This kind of discussion would be helpful to deliver the significance of why influence between Leishmania and Plasmodium was studied at the selected time point.*

 We have not examined potential cross reactivity at the T or B cell level and thank the reviewer for highlighting this alternate possibility which we have now alluded to in the discussion (new text: Discussion paragraph 4).

*Please describe the total number of repeated experiments in M&M.*

 Two independent experiments involving single *L. donovani* infection followed by drug treatment were performed. A single sequential infection experiment was performed. We have now added this information to the M&M.

*Figure 2B and elsewhere: Some data are pooled from two independent experiments, but why?* The number of animals per group does not seem increased (n=3 for uninfected controls) even by pooling.

 In Figure 2B and 3B, the data were amalgamated not pooled from two experiments, and we apologise for this error.  We conducted two experiments, but for logistical reasons and due to some technical difficulties, the time course was best represented by amalgamating the data, as now described more clearly in the figure legends (new text: Figure 2 and 3 legends). 

*Figure 6E: Why (i) C and (ii) VT look so different? They should look similar according to Figure 2. Besides, the resolution of the images is low. Images with better quality should be posted as extended data if there is a space limit in the main text.*

 We do not have a good explanation for the apparent differences in staining between control uninfected C mice and VT mice that have been rested for 7 weeks post treatment. It is possible that this reflects a  change in architecture associated with Ambisome treatment that is evident at 7 weeks but not at 4 weeks post Rx, but we feel this is unlikely.  More likely, it just reflects differences in sectioning or staining across different mouse groups (though it was a consistent finding in this mouse group).  Similarly to Figure 2, these data do however show that whereas both control and VT mice have no evidence of granulomas, these are readily apparent in VU mice (though in less abundance than mice shown in Figure 2 due to the extended time post infection).  Unfortunately we do not have higher resolution images of these sections, but feel that the main message can be conveyed from those currently provided.  

 Other minor comments:

*Page 3 and elsewhere: Infection with malaria/malaria infection is better modified to infection with Plasmodium/Plasmodium infection*

 We are happy with the reviewer’s suggestion and have made this change throughout.

*Page 3 and elsewhere: p.i to p.i.*

 We have corrected this throughout

*Page 3: About the sentence of ‘Mice were kept...’ in the paragraph of malaria infection on Procedures of M&M, I believe the 12-hour cycle is applied to all the mice even those without infection, correct? If different, please describe the light/dark cycle taken for non-infected mice.*

 The reviewer is correct and we have made this clearer in the text

*Page 5: No need to repeat the detail on F4/80 AF647 in M&M.*

 This repetition has been deleted

*Page 5: DAPI; 1μg/ml: need space between the number and the unit.*

 Thank you. This has been corrected.

*Page 8: ‘In comparison to VT mice, ...’ This does not make sense. VU should be ‘untreated’.*

 This sentence has been re-worded to address this error.

*Page 9/Figure 5A: ‘Delayed onset of thrombocytopenia after P. chabaudi infection and early recovery of platelet count…’ Not sure if the data show ‘early recovery’.*

 We agree and have modified the sentence to only refer to the delayed onset 

*Figure 1: i.v to i.v*.

 We have corrected this throughout

*Figure 4C: Not % BW ‘loss’, just % BW.*

 We have corrected this in the figure legend and provided a new Figure.

*Figure 4F: Are LDUs of VU at this time point supposed to be that low in both the spleen and liver?*

 By 11 weeks post infection (as in Figure 4F) liver parasite burdens in this mouse strain would be expected to be minimal.  The splenic parasite loads are higher, albeit slightly lower than seen in some of our previous studies, but still reflects the poorer ability of this organ to clear primary infection.  The related text as written was slightly confused, so we have re-written that for clarity.
